# Influence of Acarbose on Plasma Glucose Fluctuations in Insulin-Treated Patients with Type 2 Diabetes: A Pilot Study

**DOI:** 10.1155/2015/903524

**Published:** 2015-11-11

**Authors:** Feng-fei Li, Xiao-hua Xu, Li-yuan Fu, Xiao-fei Su, Jin-dan Wu, Chun-feng Lu, Lei Ye, Jian-hua Ma

**Affiliations:** ^1^Department of Endocrinology, Nanjing First Hospital, Nanjing Medical University, Nanjing 210012, China; ^2^Nanjing University of Chinese Medicine, Nanjing 210023, China; ^3^National Heart Research Institute Singapore, National Heart Centre Singapore, Singapore 169606

## Abstract

*Background and Aims.* To evaluate the effect of adding acarbose on glycemic excursions measured by continuous glucose monitoring system (CGMS) in patients with type 2 diabetes mellitus (T2DM) already on insulin therapy.* Materials and Methods.* This was an opened and unblended study. 134 patients with T2DM were recruited. After initial rapidly corrected hyperglycaemia by continuous subcutaneous insulin infusion (CSII) for 7 d, a 4–6-day premixed insulin titration period subsequently followed. Patients were then randomized 1 : 1 to acarbose plus insulin group or insulin therapy group for 2 weeks. CGMS was used to measure glucose fluctuations for at least 3 days after therapy cessation.* Results*. Patients in acarbose plus insulin group achieved a significant improvement of MAGE compared to that of insulin therapy only group (5.56 ± 2.16 versus 7.50 ± 3.28 mmol/L, *P* = 0.044), accompanied by a significant decrease in the incremental AUC of plasma glucose concentration above 10.0 mmol/L (0.5 [0.03, 0.9] versus 0.85 [0.23,1.4]  mmol/L per day, *P* = 0.037).* Conclusions*. Add-on acarbose to insulin therapy further improves glucose fluctuation in patients with T2DM. This study was registered with ClinicalTrials.gov registration number ChiCTR-TRC-11001218.

## 1. Introduction

Received wisdom indicated that acarbose could improve mean haemoglobin A1c (HbA1c) levels in patients with type 2 diabetes mellitus (T2DM) by reducing plasma glucose concentration [1, 2]. Furthermore, acarbose could decrease total daily insulin dose and increase the response to insulin in patients with T2DM [3, 4].

Several studies have shown that postprandial glucose (PPG) is an independent risk factor for cardiovascular disease [5]. Monnier et al. reported that acute glucose fluctuations during postprandial periods played a crucial role in oxidative stress [6]. By reducing postprandial excursions, oxidative and nitrosative stress can be diminished [7]. Studies have demonstrated that acarbose was effective in reducing postprandial glycemic excursions [8]; combined administration of acarbose with alogliptin therapy resulted in decreasing plasma glucose fluctuations [9]. Addition of acarbose to patients with T2DM who are inadequately controlled with insulin or patients initiated with insulin significantly lowers HbA1c and postprandial glucose levels [10, 11]. Recent studies have indicated that even when HbA1c is similar, glucose excursions may differ significantly [12].

We therefore performed an opened and unblended study using a continuous glucose monitoring system (CGMS) to assess the efficacy of adding acarbose to insulin therapy on plasma glucose control in patients with T2DM.

## 2. Materials and Methods

One hundred and thirty-four consecutive newly diagnosed patients with T2DM were admitted to hospital and received therapy with continuous subcutaneous insulin infusion (CSII) for initial rapid correction of hyperglycaemia. After 4–6-day premixed insulin titration period, patients were then randomized into two groups [acarbose plus insulin Isophane Protamine Recombinant Human Insulin 30/70 (premixed 30/70, twice daily) group and premixed insulin 30/70 twice-daily alone group]. The patients aged 18–75 years with a body mass index (BMI) calculated as weight in kilograms divided by the square of height in meters, 18–40 kg/m^2^, and HbA1c range 9.0–12.0%. Patients were excluded if they had acute or severe chronic diabetic complications, serious systemic disease, or poor medication compliance. Patients with known cancers, known allergies to insulin or acarbose, and an assessment by the researchers as not suitable to participate were excluded [13, 14]. The study was approved by the ethics committee of Nanjing First Hospital. Written informed consent was obtained from the patients prior to the study.

After 7-day CSII therapy, all patients achieved stable glycemic control (the fasting capillary blood glucose was less than 6.1 mmol/L and capillary blood glucose at 2 h after each of three meals was less than 8.0 mmol/L [11, 14]). Then premixed insulin 30/70 (twice daily) was administered to all patients. Initial premixed 30/70 doses were calculated as 0.4–0.5 IU/kg, and doses were subsequently adapted according to plasma glucose values obtained by self-monitoring. Investigators titrated insulin doses on an individual-patient basis at the titration algorithm (if the fasting blood glucose level was less than 4.4 mmol/L, the insulin dose was reduced 2 units; if the fasting blood glucose level was within 4.4 to 6.1 mmol/L, the insulin dose was unchanged; if the fasting blood glucose level was within 6.2 to 7.8, 7.9 to 10.0, and >10.0 mmol/L, the insulin dose was increased subsequently by 2, 4, and 6 units, resp.). Premixed insulin doses remained unchanged and recorded, if euglycemic control was achieved for two consecutive days. Patients were subsequently randomized to receive acarbose (100 mg, tid.; Glucobay, Bayer, Germany) plus premixed insulin 30/70 (twice daily) or premixed insulin 30/70 twice daily alone. Treatment was maintained for 2 weeks.

Continuous glucose monitoring (CGM) data were obtained with Medtronic Minimed CGMS Gold (Medtronic Incorporated, Northridge, USA) for at least 3 days on completion of 2 weeks randomised treatment [15]. All patients were subjected to 3 consecutive days CGMS in hospital by the specialist nurse. Shortly, the CGMS sensor was subcutaneously embedded at Day 0 around 16:00-17:00. The patients continued with the sensor for 3 consecutive days, if CGMS was going well. Subjects were instructed to keep the sensor fixed and waterproof. The study nurse inputted at least 4 calibration readings per day. At Day 3, around 16:00-17:00, subjects had the sensor removed and the CGMS data were saved by the investigator. The 24 h mean amplitude of glycemic excursions (MAGE) and other plasma glucose fluctuation parameters such as the 24 h mean blood glucose (MBG), the percentage time duration (%), and the incremental area under curve (AUC) of plasma glucose >10.0 mmol/L and <3.9 mmol/L were calculated, and hypoglycemia episodes were also recorded. MAGE was calculated for each patient by measuring the arithmetic mean of the ascending and descending excursions between consecutive peaks and nadirs for the same 24 h period; only absolute excursion values >1 SD were considered [16].

The primary endpoint was the between-group differences of 24 h MAGE. Secondary endpoints were the 24 h MBG, the AUC for hypoglycaemia (defined as sensor glucose values <3.9 mmol/L), and hyperglycaemia (sensor glucose values >10 mmol/L); the times spent in hypoglycaemia and hyperglycaemia were also analyzed.

### 2.1. Statistical Analysis

Statistical analysis was performed using SPSS software (version 17.0; SPSS, Inc., Chicago, IL). Shapiro-Wilk test was used to assess the distribution of data. Normally distributed and continuous variables are presented as mean (standard deviation, SD). Nonnormally distributed variables were presented as median (IQR) and logarithmically transformed before analysis. The independent samples* t*-test was used to compare each group difference. Bonferroni correction was followed. *P* values were two-tailed with a significance level of 5%.

## 3. Results

All subjects finished this study. Patients reached glycaemic goals in 4.3 ± 2.6 and 1.5 ± 1.4 days during the CSII period and the premixed insulin titration period, respectively.  Table 1 showed the baseline data on the 134 patients with T2DM who were randomized to the two treatment groups. The two groups did not differ significantly. All the patients completed the study.

### 3.1. Glucose Control

**Figure 1:**
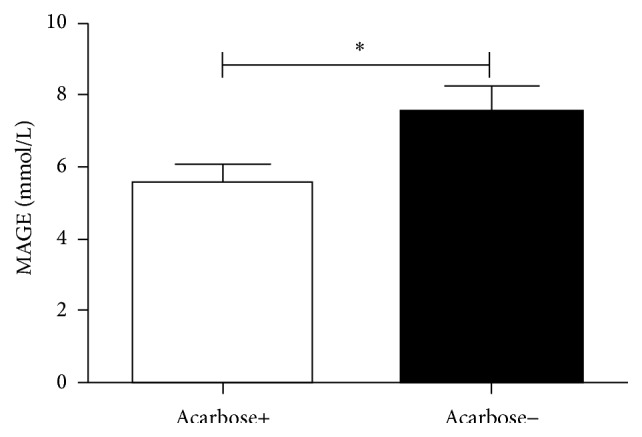


**Figure 2:**
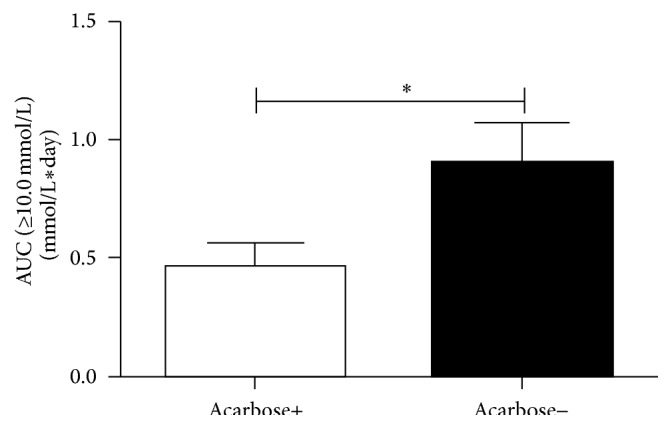


The MAGE measured by CGMS in the group of diabetes patients treated with insulin alone (7.50 ± 3.28 mmol/L) was significantly higher than the group with added acarbose (5.56 ± 2.16 mmol/L, *P* = 0.044, Figure 1).  Figure 2 shows that when acarbose was added, the incremental AUC >10 mmol/L (significant hyperglycemia) detected by CGMS was significantly decreased (0.5 [0.03,0.9] mmol/L per day, *P* = 0.037) compared to the group treated with insulin alone (0.85 [0.23,1.4] mmol/L per day).

Table 2 compared the 24 h mean glucose levels and the percentage of time of significant hyperglycemia (glucose > 10 mmol/L) and significant hypoglycemia (glucose < 3.9 mmol/L) between the two groups. There were no statistically significant differences between the acarbose group and insulin monotherapy group in the 24 h MBG, the standard deviation (SD) of the 24 h MBG, the percentage time duration of hyperglycemia (glucose > 10 mmol/L), hypoglycemia (glucose < 3.9 mmol/L), the number of hyperglycemia (glucose > 10 mmol/L) episodes, and the number of hypoglycemia excursion (glucose < 3.9 mmol/L) episodes (see Table 2).

**Figure 3:**
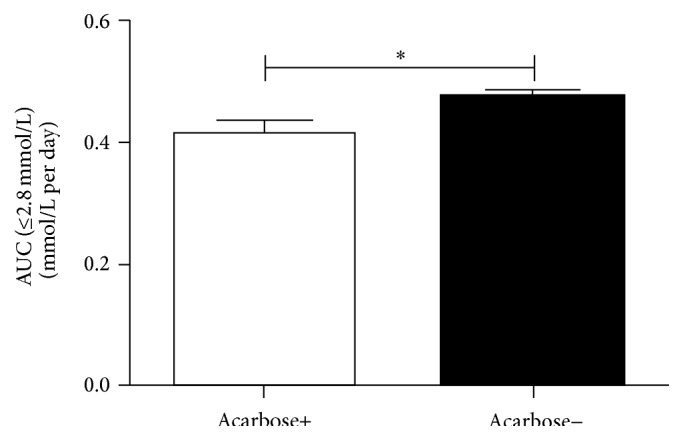


We also compared the risk of severe hypoglycemia (glucose < 2.8 mmol/L) between the two groups.  Figure 3 showed that the acarbose group had significantly less severe hypoglycemia (0.41 ± 0.20 versus 0.47 ± 0.21 mmol/L per day, *P* = 0.042). Adverse events were reported by 9 acarbose+ patients and 3 acarbose− patients, consisting of digestive disorders.

## 4. Discussion

In the current study, we observed that the addition of acarbose to insulin improves glucose fluctuation in patients with T2DM, as measured by MAGE, and reduced the risk of severe hypoglycemia.

Previous studies have shown that administration of acarbose in patients with T2DM with newly initiated insulin or established insulin therapy improved HbA1c levels. In a 20-week study, HbA1c levels in patients with a combination of acarbose and insulin had an additional improvement of 0.5% compared with a placebo group [11]. Chiasson et al. reported a mean decrease in HbA1c of 0.4% in patients already on insulin after 12-month addition of acarbose to the insulin therapy [17]. An additional improvement has also been previously described in patients treated with acarbose over a 3-year period [18]. HbA1c is very useful as evidence of long-term improvement in mean glucose in the large scale clinical studies for T2DM treatment [19, 20]. However, HbA1c does not necessarily reflect daily plasma glucose fluctuations. Patients with large glucose fluctuations around a similar mean may have implications on the risk for long-term diabetic complications [12, 21].

In the present pilot study, we expected to see a reduction of postprandial plasma glucose values in acarbose plus insulin compared with insulin monotherapy patients owing to acarbose delaying in carbohydrate absorption. Our data showed a remarkable improved MAGE with the addition of acarbose to insulin therapy compared with insulin monotherapy. The incremental AUC (glucose > 10 mmol/L) was significantly decreased when acarbose was added. This showed the inhibition of postprandial plasma glucose increases and reduction of excessive plasma glucose fluctuations when acarbose was administered. Our findings are consistent with those of previous studies in the fact that acarbose regimens have a further postprandial plasma glucose lowering effect when supplementing insulin therapy [22, 23]. In addition, we observed a significant reduction in severe hypoglycemia.

It is now believed that postprandial hyperglycemia and acute glucose fluctuations may be important as independent risk factors for cardiovascular disease in patients with onset T2DM [24], the overproduction of superoxide by the mitochondrial electron-transport chain, which induces a subsequent nitrosative stress [25], especially glucose fluctuations during postprandial periods [6]. Treatment of impaired glucose tolerant patients with long-term acarbose showed a 49% reduction in the risk of cardiovascular disease [26]. Studies also showed that acarbose improves postprandial endothelial function by improvement of postprandial hyperglycemia [27, 28]. Further studies will be needed to explore the add-on effect of acarbose for preventing complications in T2DM.

In the pilot study, newly diagnosed T2DM patients (with HbA1c > 9.0% or fasting blood glucose higher than 11.1 mmol/L) were treated with CSII therapy for 7 days for initial rapid correction of hyperglycaemia. More attention should be paid to consider the use of a CSII therapy in newly diagnosed T2DM patients. Use of CSII therapy is regarded as a safe and valuable alternative in patients with newly diagnosed T2DM. 2-3-week early CSII in patients with newly diagnosed T2DM in Chinese population achieved prolonged glycaemic remission, as well as recovery and maintenance of *β*-cell function compared with treatment with oral hypoglycaemic agents; this early insulin replacement could achieve optimum glycaemic control for 1 year [14]. In addition, the early restoration of *β*-cell function and amelioration of insulin resistance might alter the natural history of T2DM [14, 29].

In conclusion, our data suggested that adjunct acarbose administration in the short term to patients on insulin monotherapy improves glucose fluctuation in patients with T2DM.

## Figures and Tables

**Table 1:** Baseline characteristics of the study population (mean ± SD).

	Acarbose+	Acarbose−	*P* value
Number of patients (*n*)	68	66	
Male/female ratio	35/33	32/34	
Age (years)	65.75 ± 4.35	66.33 ± 7.66	0.926
Body mass (kg)	60.15 ± 10.33	62.15 ± 10.86	0.845
Body mass index (kg/m^2^)	23.63 ± 3.93	24.23 ± 2.21	0.729
HbA1c (%)	9.66 ± 1.90	9.79 ± 1.19	0.721

**Table 2:** Values of individual parameters of glucose fluctuation in the two groups at the end of the follow-up.

	Acarbose+	Acarbose−	*P* value
Insulin dose/d	36.43 ± 17.16	37.5 ± 12.40	0.891
24 h mean blood glucose (mmol/L)	8.2 ± 1.39	8.4 ± 1.64	0.711
SD of the 24 h mean blood glucose	2.34 ± 0.67	2.68 ± 1.26	0.384
Hyperglycemic episodes	3.11 ± 1.69	3.36 ± 2.17	0.739
Hypoglycemic episodes	2.09 ± 1.68	2.38 ± 1.81	0.892
Hyperglycemic time duration % (>10 mmol/L)	0.28 ± 0.25%	0.28 ± 0.19%	0.963
Hypoglycemic time duration % (<3.9 mmol/L)	0.05 ± 0.08%	0.07 ± 0.12%	0.629
